# Clinical judgement, case complexity and symptom scores as predictors of outcome in depression: an exploratory analysis

**DOI:** 10.1186/s12888-020-02532-0

**Published:** 2020-03-16

**Authors:** M. Smith, B. Francq, A. McConnachie, K. Wetherall, A. Pelosi, J. Morrison

**Affiliations:** 1 NHS Greater Glasgow and Clyde, Glasgow, UK; 2 Institute of Statistics, Biostatistics and Actuarial Sciences, Université Catholique de Louvain, Ottignies-Louvain-la-Neuve, Belgium; 3 Robertson Centre for Biostatistics, University of Glasgow, Glasgow, UK; 4 The Priory Hospital, Glasgow, UK; 5 Senate Office, University of Glasgow, Glasgow, UK

**Keywords:** Depression, Anxiety, Recovery, Primary care, Outcomes, Drop-out

## Abstract

**Background:**

Clinical guidelines for depression in adults recommend the use of outcome measures and stepped care models in routine care. Such measures are based on symptom severity, but response to treatment is likely to also be influenced by personal and contextual factors. This observational study of a routine clinical sample sought to examine the extent to which “symptom severity measures” and “complexity measures” assess different aspects of patient experience, and how they might relate to clinical outcomes, including disengagement from treatment.

**Methods:**

Subjects with symptoms of depression (with or without comorbid anxiety) were recruited from people referred to an established Primary Care Mental Health Team using a stepped care model. Each participant completed three baseline symptom measures (the Personal Health Questionnaire (PHQ), Generalised Anxiety Disorder questionnaire (GAD) and Clinical Outcomes in Routine Evaluation (CORE-10)), and two assessments of “case complexity” (the Minnesota-Edinburgh Complexity Assessment Measure (MECAM) and a local complexity assessment). Clinician perception of likely completion of treatment and patient recovery was also assessed. Outcome measures were drop out and clinical improvement on the PHQ.

**Results:**

298 subjects were recruited to the study, of whom 258 had a sufficient dataset available for analysis. Data showed that the three measures of symptom severity used in this study (PHQ, GAD and CORE-10) seemed to be measuring distinct characteristics from those associated with the measures of case complexity (MECAM, previous and current problem count). Higher symptom severity scores were correlated with improved outcomes at the end of treatment, but there was no association between outcome and complexity measures. Clinicians could predict participant drop-out from care with some accuracy, but had no ability to predict outcome from treatment.

**Conclusions:**

These results highlight the extent to which drop-out complicates recovery from depression with or without anxiety in real-world settings, and the need to consider other factors beyond symptom severity in planning care. The findings are discussed in relation to a growing body of literature investigating prognostic indicators in the context of models of collaborative care for depression.

## Background

Clinical guidelines for the management of depression recommend the use of clinical outcome measures and stepped care models in routine care [1], but it can be difficult for health services to match individual patients to the most appropriate intervention, and outcomes are often poor. Large studies in the UK and the USA have found that as few as 22% of patients receive adequate care [2], that half of patients show no response to treatment [3]; and that only 30% of cases conclude with a “planned ending” [4].

“Collaborative care” is an approach to the delivery of evidence-based mental health care for primary care patients which seeks to address these problems [5]. It includes three core elements: team-based multidisciplinary care delivery, implementation of a stepped care model (in which the intensity of care is stepped up or down depending on response to treatment), and the systematic collection of clinical outcome data to inform decisions about treatment [6–8]. The data chosen to comprise such “measurement-based care” conventionally measures depressive symptoms, adherence to treatment and side effects [9].

Guidance on depression care issued by the UK National Centre for Health and Care Excellence (NICE) recognises that “a wide range of biological, psychological and social factors, which are not captured well by current diagnostic systems, have a significant impact on the course of depression and the response to treatment” [10].

Researchers have called for investigation of treatment factors that extend beyond the choice of therapies implemented [3]. These influences include service-side factors, such as the attitude and aptitude of the treating clinicians [11]. Adherence to treatment is improved by good communication, management of expectations, patient activation and shared decision making [12–14]. Unplanned drop-out from care is an important adverse outcome, since the opportunity to modify treatment to better meet patient needs has been lost. Although important, none of these contextual influences are captured in the measures of symptom severity conventionally used to deliver collaborative care [15].

In practice, clinical assessments are based on case formulations that consider social, developmental and psychological factors, and take place in teams which should have the ability to reflect on their own style of engagement with patients.

Two forms of assessment were investigated: symptom severity measures (using the PHQ, GAD and CORE-10) and case complexity measures (using the MECAM and a bespoke case complexity instrument). The measures are described in more detail in the Methods section. This study aimed to investigate the feasibility of measuring a broader range of contextual factors when planning care for people with depression (with or without comorbid anxiety), and to assess the association of those factors with clinical outcomes, including drop out from treatment.

## Methods

The service described in this paper has implemented a stepped care programme of treatment for depression in keeping with NICE guidance since its inception in 2004. The service model is described in detail elsewhere [16]. It includes the use of routine symptom severity measures (PHQ and GAD) to guide the use of brief psychological therapies by mental health clinicians working in a Primary Care Mental Health Team, but supervised from secondary care. Patients can access guided self-help, antidepressant medicines and/or (typically) 4–6 sessions of therapies such as Interpersonal Therapy and Cognitive Behavioural Therapy. Patients not responding to these interventions have prompt access to psychology, psychotherapy and/or psychiatry assessments, and ultimately to longer-term pharmacological and psychological therapies in secondary care.

Subjects with symptoms of depression defined as a score of five or more on the PHQ were recruited from people referred to a Primary Care Mental Health Team based in a town near a large urban centre in Scotland. They were included in the study whether or not they had comorbid anxiety symptoms as measured by the GAD. The PCMHT provides a service to patients aged 18 years or more, who are referred by their GP with a new case of depression, anxiety, low mood, adjustment disorder or some combination of these problems. A “new” case was defined as having been well for 6 months prior to the onset of current mood problems, or having been referred within 2 months of onset of treatment in primary care of the current episode.

People presenting with a primary alcohol problem, a primary drug problem, depression as part of bipolar affective disorder, and those with a terminal illness, medical or psychiatric emergencies or current psychosis were referred for more appropriate forms of care and hence excluded from this study, in keeping with standard local NHS procedures.

Before the first appointment, an information pack was sent to every patient outlining the clinical service they would receive, and inviting them to participate in this research study, with an information sheet and copy of the consent form. At the first appointment, clinicians reviewed the clinical and research information with the patient and invited them to consent to the research programme. If the patient chose not to consent, the appointment continued with “treatment as usual” as described in existing treatment protocols. If the patient did consent to participate, the following assessments were completed before continuing treatment as described in existing protocols.

The assessment was in four parts:

1. Collection of demographic information (age, gender, ethnicity, postcode and employment status)
2. Completion of clinical symptom severity measures at each visit: the Personal Health Questionnaire (PHQ), Generalised Anxiety Disorder questionnaire (GAD) and Clinical Outcomes in Routine Evaluation (CORE-10)
3. Completion of complexity measures at initial visit only: the Minnesota-Edinburgh Complexity Assessment Measure (MECAM) and the local complexity measure.
4. The clinician’s view about whether the patient was (a) likely to attend further treatment visits, and (b) whether they felt the patient’s symptoms were likely to improve with an intervention of 5 sessions or less. These “engagement and prognosis” questions were asked at the initial visit only.

The PHQ is a self-reported, nine item questionnaire for the assessment of low mood. It incorporates DSM-IV depression diagnostic criteria, and scoring is based on the frequency of symptoms during the previous 2 weeks. It can be administered repeatedly to track the clinical course during treatment. Scores greater than 4, 9, 14 and 19 or more represent mild, moderate, moderately severe and severe depression respectively [17]. A reduction in score of five points or more is generally considered to represent a clinically significant improvement [17, 18].

The GAD is a seven item self-reported questionnaire used for assessment of generalised anxiety disorder that may be completed by the clinician or patient. Like the PHQ, scoring is based on the frequency of symptoms in the previous 2 weeks. Scores greater than 4, 9 and 14 points or more represent mild, moderate or severe anxiety [19].

The 34-item CORE-OM Clinical Outcomes in Routine Evaluation tool is a generic measure of psychological distress which covers a range of presenting problems [20]. The CORE-10 is a 10-item version of the full measure, and is used as a screening tool and outcome measure when the CORE-OM is considered too long for routine use. The measure includes two questions each about anxiety and depression, and one question each about trauma, physical problems and risk to self. A further three items enquire about day to day functioning, close relationships and social relationships. The clinical cut-off score for general psychological distress is 11 [21].

The Minnesota Edinburgh Complexity Assessment Method (MECAM [22]) is a clinician-rated measure which was designed to encourage a holistic assessment of patient needs, and initially developed for use in primary care in the UK. It asks 11 questions in four domains: Health and Wellbeing, Social Environment, Health Literacy and Communication and Action [23]. The items are scored on a four-point scale, as shown in Appendix 2.

A local indicator of case complexity was developed that could be completed by the assessing clinician at the end of the first visit. The indicator used a range of factors derived from a review of 20 team clinical case notes and relevant literature [24–27]. Indicators of case complexity comprised information that would be routinely collected as part of a standard psychiatric assessment, and included 12 “current difficulties” (such as childcare, money or housing problems), 15 “past difficulties” (such as a history of physical, sexual or emotional abuse, or parental alcohol or drug dependence), current receipt of benefits, alcohol consumption, smoking, presence of a long-term health condition, or presence of two or more health conditions. These measures were collated into counts. The full list of questions in the case complexity measure is shown in Appendix 1.

In addition, the treating clinician was asked to state at baseline whether they thought that “the patient’s problems will improve with an intervention of 5 sessions or less”, and whether the clinician thought that “this patient is more than 50% likely to attend their next appointment with you?”

All clinical information was held securely on NHS systems in keeping with NHS Greater Glasgow and Clyde policies on information governance.

Data for analysis was anonymised by removing names, date of birth, and the Community Health Index number (CHI; the unique identifier for all patients in the NHS in Scotland) from all research records. The CHI number was replaced with a personal research ID number independent of CHI. The key linking CHI and research database identification numbers was held securely in keeping with standard protocols.

Baseline characteristics of the participants such as gender, age, ethnicity and health conditions, as well as baseline complexity and symptom severity measures are presented descriptively. Deprivation was measured using the Scottish Index of Multiple Deprivation, the official tool used by Scottish Government to identify areas of poverty and inequality in Scotland [28]. Associations between complexity measures and symptom severity measures at baseline were examined.

An exploratory Principal Component Analysis (PCA) was performed to assess the pattern of associations between the complexity measures and symptom severity measures at baseline. Wilcoxon-Mann-Whitney tests were used to compare complexity measures and symptom severity measures between groups defined by the clinician’s two “engagement and prognosis” questions at the first visit.

We first investigated factors associated with the likelihood of drop out from the service. Drop out was defined as failure to return for treatment visits after the initial assessment visit. Drop out was summarised in relation to baseline patient characteristics, complexity and symptom severity measures, and the clinician assessment of whether the patient was likely to disengage, and associations were assessed with Fisher’s Exact Test *p*-values. Univariate and multivariable logistic regression models were also used to explore these associations.

We then investigated the factors associated with treatment response, defined as achieving at least a 5 point improvement in PHQ score, amongst those who attended at least one treatment visit. This is compatible with the “reliable change index” for the PHQ used by other researchers [3]. Descriptive statistics, and Fisher’s Test p-values are reported. Univariate and multivariable logistic regression models were also performed.

## Results

### Baseline characteristics

927 referrals were made to the service during the study period, and 576 patients attended at least one appointment, making them eligible to join the study. Of that number, 298 subjects were recruited to the study, of whom 258 had an adequate dataset available for analysis.

Participant characteristics at baseline are summarised in Table 1. Participants were 159 women and 99 men, with a mean age of 38.6 years. Participants were drawn from each of the five deprivation quintiles in the Scottish Index of Multiple Deprivation. The distribution in this study was broadly similar to that of the catchment area. Thirty-two percent lived in the most deprived quintile, 15% in the second most deprived, 19% in the third most deprived, 10% in the fourth most deprived and 25% in the least deprived quintile. Our study population was slightly less deprived than the population in the overall catchment area of the study with 26, 15, 20, 15 and 23% in the most to least deprived quintiles respectively [29]. Nineteen percent were receiving one or more of the following benefits: job-seeker’s allowance, employment support allowance, disability living allowance, or free school meals for children.

**Table 1:** Baseline characteristics

		Male	Female	All
N		99	159	258
Age (years)	Mean (SD)	39.5 (13.4)	38.0 (12.4)	38.6 (12.8)
Current smoker	N (%)	30 (30%)	31 (19%)	61 (24%)
Current Antidepressant Use	N (%)	59 (60%)	81 (51%)	140 (54%)
Complexity Measures				
Past Difficulties Count	Mean (SD)	3.0 (2.5)	3.0 (2.2)	3.0 (2.3)
Current Difficulties Count	Mean (SD)	4 (2.1)	4.4 (2)	4.2 (2)
MECAM	Mean (SD)	18.9 (5.7)	19.1 (5)	19.0 (5.3)
Symptom Scores				
PHQ	Mean (SD)	16 .0 (5.6)	15.9 (5.6)	16.0 (5.6)
GAD	Mean (SD)	13.5 (4.8)	13.8 (4.7)	13.7 (4.7)
CORE-10	Mean (SD)	21.2 (6)	21.5 (6.4)	21.3 (6.2)
Clinician Assessments				
Do you think this patient is more than 50% likely to attend their next appointment with you?				
	“Yes” N (%)	86 (87%)	147 (92%)	233 (90%)
Do you think this patient’s problems will improve with an intervention of 5 sessions or less?				
	“Yes” N (%)	71 (72%)	127 (80%)	198 (77%)

Ten percent of subjects reported that they drank to an “increased-risk” or “higher-risk” level as defined by NICE guidance (at that time, above 21 units of alcohol per week for men and 14 units per week for women) [30]. 24% of subjects reported that they currently smoked cigarettes. Sixty-four percent of participants described themselves as “healthy”, with 22% reporting at least one long-term health condition and 14% having two or more long-term conditions. Just over half of participants said that they were both prescribed, and taking, antidepressant medicine.

None of the differences between male and female patients were statistically significant.

### Association between baseline symptoms severity measures and case “complexity”

There were moderate, but highly significant associations between the complexity measures and symptom severity measures at baseline (Table 2).

**Table 2:** Spearman correlation coefficients between baseline complexity measures and symptom severity measures, with *p*-values

		Complexity Measures		
		Past Difficulties	Current Difficulties	MECAM
Symptom severity measures	PHQ	r = 0.24 *p* < 0.001	r = 0.26 *p* < 0.001	r = 0.41 *p* < 0.001
	GAD	r = 0.17 *p* = 0.005	r = 0.15 *p* = 0.016	r = 0.25 *p* < 0.001
	CORE-10	r = 0.28 *p* < 0.001	r = 0.22 *p* < 0.001	r = 0.35 *p* < 0.001

There were statistically signifiant associations between each of the symptom severity measures (PHQ-GAD (0.65), PHQ-CORE (0.72), GAD-CORE (0.72)) and each of the complexity measures (MECAM-Past (0.38), MECAM-Current (0.53), Past-Current (0.23)), all *p* < 0.001).

**Fig. 1:**
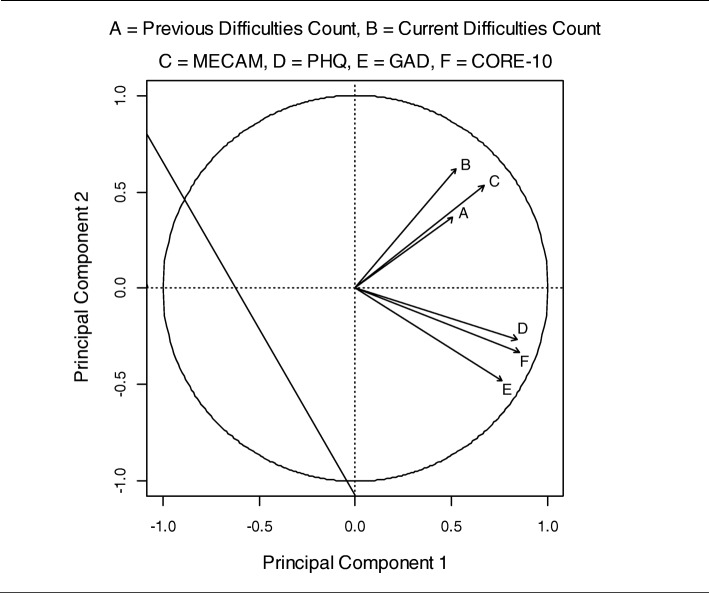
Correlations between input variables and the first two components from a Principal Components Analysis of complexity measures (Previous Problem Count, Current Problem Count, MECAM) and symptom severity measures (PHQ, GAD, CORE-10) at baseline

An exploratory Principal Components Analysis of complexity measures and symptom severity measures at baseline identified two components that explained 49.7 and 20.3% of the total variation, respectively; Fig. 1 suggests that the first principal component measures general case severity, being positively correlated with all six scores. The second component differentiates between the complexity measures (positively correlated) and symptom severity measures (negatively correlated). This interpretation of the second principal component, coupled with the observation that the complexity and outcomes measures show a clear separation in terms of their patterns of correlations with the two principal components, indicate that the two groups of measures are describing distinct features of patients’ clinical condition at baseline.

Complexity scores were also correlated with clinician views on prognosis at first assessment. Patients whose clinicians thought they were likely to improve with an intervention of 5 sessions or less had significantly lower problem counts and MECAM scores, and lower PHQ and CORE-10 scores (Table 3). Patients who the clinician thought were likely to continue with treatment had a lower number of current problems (though not previous problems) and lower MECAM scores. There were no significant associations between clinician assessments about probable attendance and any of the symptom severity scores at baseline.

**Table 3:** Mean (SD) of complexity measures and symptom scores in relation to clinician assessments, with p-values from Wilcoxon-Mann-Whitney tests

	Do you think this patient’s problems will improve with an intervention of 5 sessions or less?		
	Yes	No	p-value
N	198	60	
Complexity Measures			
Previous Problem Count	2.7 (2.2)	4.0 (2.5)	*p* < 0.001
Current Problem Count	4.0 (2.0)	4.8 (2.1)	*p* = 0.013
MECAM	18.3 (4.7)	21.5 (6.2)	*p* < 0.001
Symptom Scores			
PHQ	15.4 (5.6)	17.7 (5.4)	*p* = 0.009
GAD	13.5 (4.7)	14.4 (4.7)	*p* = 0.161
CORE-10	20.8 (6.2)	23.3 (5.9)	*p* = 0.004
	Do you think this patient is more than 50% likely to attend their next appointment with you?		
	Yes	No	p-value
N	233	25	
Complexity Measures			
Previous Problem Count	3.0 (2.3)	3.0 (2.2)	*p* = 0.964
Current Problem Count	4.1 (2.0)	4.8 (1.8)	*p* = 0.061
MECAM	18.7 (5.0)	21.8 (6.9)	*p* = 0.040
Symptom Scores			
PHQ	15.8 (5.5)	17.1 (6.3)	*p* = 0.380
GAD	13.6 (4.7)	14.6 (4.8)	*p* = 0.270
CORE-10	21.2 (6.3)	23.1 (5.6)	*p* = 0.111

### Association between baseline measures and drop out from treatment

Being a current smoker was associated with a significantly increased likelihood of drop out from treatment, but age, gender and antidepressant use were not (Table 4). Neither the symptom severity measures nor the complexity measures were associated with dropping out from treatment. The clinician assessment about who would drop out of care was significantly associated with subsequent drop out. Although clinicians only predicted 25 subjects to be unlikely to attend the next visit, 80% of their predictions were correct. Logistic regression analyses (Table 5) broadly supported these findings.

**Table 4:** Association between baseline patient characteristics, complexity and symptom severity measures, and clinician assessments, and patient drop out and, for those who engaged, treatment response. Drop out defined as failure to attend treatment visits after the initial assessment. N total is everyone who attended the initial visit, and N engaged is the participants that attended the initial visit and at least one treatment visit. Treatment response defined as achievement of at least a 5 point improvement in PHQ score at the last attended visit up to the 5th treatment visit. P-values from Fisher’s Exact Tests

		Drop out			PHQ Response		
		N Total	N Dropout (%)	p-value	N Engaged	N Improvement (%)	p-value
	All	258	99		159	98	
Age (years)	18–29 30–44 45+	72 104 82	33 (46%) 35 (34%) 31 (38%)	*p* = 0.257	39 69 51	30 (77%) 43 (61%) 25 (47%)	*p* = 0.026
Gender	Male Female	99 159	45 (46%) 54 (34%)	*p* = 0.067	54 105	35 (65%) 63 (60%)	*p* = 0.608
Current smoker	Yes No	61 197	33 (54%) 66 (34%)	*p* = 0.006	28 131	22 (79%) 76 (58%)	*p* = 0.054
Current AD Use	Yes No	140 118	55 (39%) 44 (37%)	*p* = 0.798	85 74	56 (66%) 42 (57%)	*p* = 0.256
Prev Prob Count	Low (≤2) High (≥3)	129 129	49 (38%) 50 (39%)	*p* = 1.000	80 79	47 (59%) 51 (65%)	*p* = 0.515
Curr Prob Count	Low (≤4) High (≥5)	149 109	60 (40%) 39 (39%)	*p* = 0.518	89 70	56 (63%) 42 (60%)	*p* = 0.744
MECAM	Low (≤18) High (≥19)	125 133	49 (39%) 50 (38%)	*p* = 0.799	76 83	43 (57%) 55 (66%)	*p* = 0.254
PHQ	None/Mild/Moderate Moderate severe Severe	98 79 125	41 (42%) 27 (34%) 31 (38%)	*p* = 0.574	57 52 50	26 (46%) 34 (65%) 38 (76%)	*p* = 0.005
GAD	Minimum/Mild Moderate Severe	54 79 125	23 (43%) 33 (42%) 43 (34%)	*p* = 0.446	31 46 82	15 (48%) 19 (41%) 64 (78%)	*p* < 0.001
CORE-10	Low/Mild/Moderate Moderate severe Severe	97 59 102	43 (44%) 15 (25%) 41 (40%)	*p* = 0.054	54 44 61	29 (54%) 25 (57%) 44 (72%)	*p* = 0.096
Clinician assessment^(a)^	Yes No	233 25	79 (34%) 20 (80%)	*p* < 0.001			
Clinician assessment^(b)^	Yes No				129 30	80 (62%) 18 (60%)	*p* = 0.838

**Table 5:** Association between baseline patient characteristics, symptom scores, complexity scores, and clinician’s assessment^(a, b)^, and treatment response, defined as achievement of a 5 point reduction in PHQ-9 score at last attended visit up to the 5th treatment visit, and drop out form the service, defined as failure to return for treatment after the initial assessment visit. Data analysed using univariate and multivariable logistic regression models. Results presented as odds ratios, with 95% confidence intervals and p-values. Best-fitting multivariable models found by backward selection from all predictors

Predictor	Effect	Drop out from service		Achievement of 5 point reduction in PHQ-9	
		Univariable	Multivariable	Univariate	Multivariable
		OR (95% CI), p-value	OR (95% CI), *p*-value	OR (95% CI), p-value	OR (95% CI), p-value
Age	per 10 years	1.03 (0.85, 1.26), *p* = 0.756		0.72 (0.54, 0.94), *p* = 0.017	0.71 (0.52, 0.95), *p* = 0.022
Gender	Female vs. Male	0.62 (0.37, 1.03), *p* = 0.066		0.81 (0.41, 1.6), *p* = 0.555	
Current Smoker	Yes vs. No	2.34 (1.31, 4.22), *p* = 0.004	2.49 (1.3, 4.81), *p* = 0.006	2.65 (1.06, 7.6), *p* = 0.048	
Current AD Use	Yes vs. No	1.09 (0.66, 1.81), *p* = 0.742		1.47 (0.77, 2.81), *p* = 0.239	
Prev Prob Count	per problem	1.02 (0.92, 1.14), *p* = 0.701		1.13 (0.97, 1.32), *p* = 0.131	
Curr Prob Count	per problem	0.94 (0.82, 1.06), *p* = 0.299		1.02 (0.87, 1.2), *p* = 0.82	
MECAM	per point	0.97 (0.93, 1.02), *p* = 0.275	0.93 (0.88, 0.98), *p* = 0.01	1.03 (0.97, 1.1), *p* = 0.333	
PHQ-9	per point	1 (0.95, 1.04), *p* = 0.953		1.13 (1.07, 1.21), *p* < 0.001	1.13 (1.07, 1.21), *p* < 0.001
GAD	per point	0.96 (0.91, 1.01), *p* = 0.131		1.17 (1.09, 1.27), *p* < 0.001	
CORE-10	per point	0.98 (0.94, 1.02), *p* = 0.416		1.09 (1.04, 1.16), *p* = 0.002	
Clinician assessment^(a)^	No vs. Yes	7.8 (3.03, 24.13), *p* < 0.001	8.59 (3.16, 27.94), *p* < 0.001	–	
Clinician assessment^(b)^	Yes vs. No	–		1.09 (0.47, 2.43), *p* = 0.838	

### Association between baseline measures and clinical improvement

There was a high attrition rate over time, with 159 of 258 participants returning for a second appointment, and only 39 completing the 5th treatment visit (Table 6).

**Table 6:** Mean (SD) symptom severity scores (PHQ, GAD, CORE-10) from the first 5 treatment visits (Visit 1 was the initial assessment). P-values are also included as a comparison of mean symptom severity at each visit compared with the previous visit (where applicable) using paired t-tests

	Visit 1	Visit 2	Visit 3	Visit 4	Visit 5
N	258	159	99	63	39
PHQ	16 (5.6)	11.8 (6), *p* < 0.001	10.7 (5.9), *p* < 0.001	9.6 (5.9), *p* = 0.003	10.7 (6.3) *p* = 0.369
GAD	13.7 (4.7)	10.3 (5.4), *p* < 0.001	9.7 (5.1), *p* = 0.002	8.6 (5.3), *p* < 0.001	9.1 (5.6), *p* = 0.360
COR	21.3 (6.2)	16.8 (7.7), *p* < 0.001	15.7 (7), *p* < 0.001	13.8 (8.2), *p* < 0.001	15.2 (9.1), *p* = 0.233

Clinical improvement was defined as the achievement of a 5-point or greater reduction in PHQ score, at the last visit attended after the initial assessment, up to the 5th treatment visit, therefore the 99 participants who did not attend at least one treatment visit after the initial assessment were not included in the analyses looking at clinical improvement. Of the 159 participants that attended at least one treatment visit after the initial assessment, 98 achieved at least a 5-point improvement in PHQ score (Table 4).

Younger patients who engaged with the service were more likely to show an improvement in PHQ scores, but there was no difference by gender, or by antidepressant use (Table 4). None of the complexity measures showed any association with treatment response, but there was an association with baseline PHQ and GAD, with more severely affected patients showing the greatest improvements. Interestingly, clinicians were unable to predict which patients were most likely to improve within 5 treatment sessions. On multivariable regression, only baseline PHQ remained a significant predictor of treatment response, amongst the baseline symptom severity measures. Otherwise, only younger age remained as an independent predictor of improvement in PHQ scores.

## Discussion

This exploratory study sought to investigate the associations between symptom severity scores and a range of contextual factors on clinical outcomes, including drop out from treatment. It was based on observation of a routine clinical sample, conducted in a team providing care in a collaborative, stepped care model. The patient group were a typical UK cohort in terms of demographics and symptom severity.

The use of measurement-based care to improve outcomes depends on three premises: firstly, that the measurements accurately assess relevant influences on treatment, secondly that they can inform meaningful choices between treatment options, and thirdly that they can facilitate the early identification of potential harm [9]. Such harm might include clinical deterioration, emerging risks to safety or the likelihood of dropping out of treatment altogether.

### Main findings

The majority of patients presented with “moderately severe” or “severe” depression, which is consistent with other studies of “low intensity” interventions [31], including the Second UK National Audit of psychological therapies [3]. Recruitment to the study was 45% of eligible subjects, comparable to other work in Improving Access to Psychological Therapies (IAPT) settings in England [32]. Drop-out rates were high, with 61% of patients attending a second appointment, and only 15% attending for five appointments. This is higher than drop-out rates of 20–40% observed in other mental health settings [33, 34], though it should be noted that the typical number of planned care visits in UK primary care mental health settings is only about six [4]. Nonetheless, the mean improvement scores on the PHQ for patients who adhered to treatment was clinically significant and statistically significant between all treatment visits apart from between the 4th and 5th treatment visit. Symptom severity scores showed the biggest decrease between the first and second visit, with treatment continuing beyond four visits associated with less improvement.

The three measures of symptom severity (PHQ, GAD and CORE-10) were significantly correlated with each other, as were the three measures of case complexity (MECAM, previous problem count and current problem count). The symptom severity and complexity measures were also correlated with each other. However principal components analysis suggested that symptom and complexity measures at intake were associated with different aspects of patient characteristics at baseline.

Neither symptom severity nor complexity measures were associated with drop out from treatment when considered individually. On a multivariable analysis, current smoking status and the MECAM were associated with drop out. The effect with MECAM was small (OR 0.93). This is a surprising finding, since the complexity measures included socioeconomic characteristics such as unemployment or being in receipt of benefits; these would usually be considered adverse prognostic indicators. The absence of an association between experience of past adversity and drop out from treatment was surprising, given the extent to which Adverse Childhood Experiences (ACEs) are known to increase the prevalence of depression and the risk of poor outcomes in treatment [35, 36].

This study confirms clinician’s inability to predict the likely outcome of treatment, and the importance of baseline illness severity in predicting improvement. Although the MECAM had a modest association with drop out from treatment, clinician impression was more influential. We found that clinicians could predict with some accuracy who would drop out of treatment. This effect remained influential (OR 8.59) in the multivariable analysis, although one third of patients who dropped out of treatment were not identified by the clinician. The accuracy of the clinician assessment in relation to drop out of treatment may be an early indication of difficulties in the therapeutic relationship (making drop-out more likely), or perhaps reflects a complex qualitative intuition expressed by clinicians which is not fully captured by the quantitative measures.

Baseline scores for the PHQ and GAD predicted treatment response, with more severe scores being more likely to show an improvement in PHQ of at least 5 points. On the multivariable analysis, only PHQ and younger age were associated with improvement. Clinician judgement showed no ability to determine who was most likely to improve with treatment.

Although baseline characteristics may be associated with outcome from treatment, research to date has not been able to define clear “steps” which stepped care models might use. Two recent meta-analyses using individual patient level data found that outcomes after Cognitive Behavioural Therapy (CBT) were independent of baseline severity for both CBT [37] and antidepressants or CBT [38]. The optimal treatment choices at each step may not be clear [39]. Treatment guidelines therefore differ: guidance from the National Institute for Health and Care Excellence (NICE) in England reserves antidepressant treatment for more severe depression (PHQ score 18+) [1], whereas American guidelines for the treatment of depression in primary care advocates both antidepressant and psychotherapy for patients with a PHQ score above 10 [40].

Pragmatic trials of smaller numbers of prognostic indicators [41, 42] have confirmed the importance of baseline severity of depression and anxiety, as well as other factors such as age, gender, living alone and marital status.

### Strengths and limitations

One strength of the study was that it took place in a routine care setting, since participants in randomised controlled trials for depression and anxiety may not be representative of those who attend primary and psychiatric care clinics [39].

However, these advantages are also associated with some weaknesses. Antidepressants and psychological therapy were used in varying combinations for different patients. The study took place in a team designed to deliver “brief interventions”, which in a UK primary care mental health setting are typically of 6–10 sessions [4, 43].

Epidemiological studies suggest that the mean duration of a depressive episode is about 4 months [2]. In trials both natural remission (43% within 6 months) and placebo response rates (49%) in primary care trials are high [15, 44]; but this uncontrolled study was not able to investigate their effects.

Studies of Adverse Childhood Experiences show that not all negative life experiences have the same impact on later-life outcomes [45], yet in this study there was no adjustment for the severity or chronicity of those experiences. Likewise, no enquiry was made about potential protective factors, even though these may influence outcomes [46, 47].

## Conclusions

This study highlights the importance of drop-out from care as an important therapeutic consideration. Drop-out has been found to be more likely early in treatment, particularly after the second visit. Strategies to minimise drop out include attending to health literacy and patient expectations of treatment [48, 49], establishing a therapeutic alliance [50], and of “person-centred” approaches to care and shared decision making [51].

King commented that “real-world data show that outcomes from psychotherapy are messy and difficult to predict” [52]. This observational study confirms that condition severity is the best indicator of outcome, and finds that clinician judgement may be a useful predictor of potential drop out from treatment. Further research into the characteristics of clinician prediction might usefully complement other measures used in collaborative care.

## Supplementary information

**Additional file 1.** Appendix 1: Local “Complexity” measures. Appendix 2: MECAM items.

## Data Availability

The datasets used and/or analysed during the current study are available from the corresponding author on reasonable request.

## Abbreviations

CBT: Cognitive Behavioural Therapy
CHI: Community Health Index number (a unique patient identifier for the NHS in Scotland)
CORE: Clinical Outcomes in Routine Evaluation
DSM-IV: American Psychiatric Association Diagnostic and Statistical Manual, Fourth Edition
GAD: Generalised Anxiety Disorder Questionnaire
IAPT: Improving Access to Psychological Therapies
MECAM: Minnesota-Edinburgh Complexity Assessment Measure
NHS: (UK) National Health Service
NICE: (UK) National Centre for Health and Care Excellence
OR: Odds Ratio
PCA: Principal Component Analysis
PCMHT: Primary Care Mental Health Team
PHQ: Personal Health Questionnaire
SD: Standard Deviation
SIMD: Scottish Index of Multiple Deprivation
