# Supplementary material for: Clinical judgement, case complexity and symptom scores as predictors of outcome in depression: an exploratory analysis
Source: BMC Psychiatry. 2020 Mar 16;20:125. doi: 10.1186/s12888-020-02532-0 (PMC7076946; doi:10.1186/s12888-020-02532-0)
Supplement: Supplementary file 1 — Additional file 1. Appendix 1: Local “Complexity” measures. Appendix 2: MECAM items. [file 12888_2020_2532_MOESM1_ESM.docx]

**Appendix 1: Local “Complexity” measures**

Each of the following items was rated “yes”, “no”, or “unknown”.

**A. Past history of:**

1. Being bullied in school or in the workplace.

2. Being sexually abused

3. Being physically abused

4. Being emotionally abused

5. Being neglected

7. Social work/child protection involvement in childhood

8. Adopted, fostered or in care during childhood

9. Personal alcohol harmful use or dependence

10. Parental alcohol harmful use or dependence

11. Personal drug harmful use or dependence

12. Parental drug harmful use or dependence

13. Family history of diagnosed mental illness

14. Family history of suicide

15. Previous personal history of depression/anxiety

**B. Current problems:**

1. Childcare problems

2. Money problems

3. Relationship problems with partner(s) and ex-partner(s)

4. Relationship problems with family/friends including isolation

6. Being a Vulnerable Adult

5. Work-related stresses

6. Unemployment and seeking work

7. Bereavement and/or other current significant losses

8. Grievance (perceived injustice that can't be resolved)

9. Active issues involving police, court, prison

10. Housing problems

11. Being a carer

12. Children < 18 at home

**Appendix 2: MECAM items**

**A. Health and Wellbeing**1. Thinking about your patient's physical health needs, are there any symptoms or problems you are unsure about that require further investigation?

a. No identified areas of uncertainty or problems already being investigated
b. Mild vague physical symptoms or problems; but do not impact on daily life or are not of concern to patient
c. Moderate to severe symptoms or problems that impact on daily life
d. Severe symptoms or problems that cause significant impact on daily life
e. Not sure

2. Are the patient's physical health problems impacting on their mental well-being?

a. No identified areas of concern
b. Mild impact on wellbeing, eg "feeling fed up", "reduced enjoyment")
c. Moderate to severe impact on mental well-being and preventing enjoyment of usual activities
d. Severe impact upon mental well-being and preventing engagement with usual activities
e. Not sure

3. Are there any problems with your patient's lifestyle behaviours (alcohol, drugs, diet, exercise) that are impacting on mental wellbeing?

a. No identified areas of concern
b. Some mild concern of potential negative impact on wellbeing
c. Moderate to severe impact on patient's wellbeing, preventing enjoyment of usual activities
d. Severe impact on patient's mental health with additional potential impact on others
e. Not sure

4. Do you have any other concerns about your patient's mental wellbeing? How would you rate their severity and impact on the patient?

a. No identified areas of concern
b. Mild problems- don't interfere with function
c. Moderate to severe problems that interfere with function
d. Severe problems impairing most daily functions
e. Not sure

**B. Social Environment**

1. How would you rate their home environment in terms of safety and stability (including domestic violence, insecure tenancy, neighbour harassment)?

a. Consistently safe, supportive, stable. No identified problems
b. Safe, stable, but with some inconsistency
c. Safety/stability questionable
d. Unsafe and unstable
e. Not sure

2. How does unemployment (or risk of unemployment), work or other daily activities (eg caring) impact on the patient's wellbeing?

a. No identified problems or perceived positive benefits
b. Some general dissatisfaction but no concern
c. Contributes to low mood or stress at times
d. Severe impact on poor mental wellbeing
e. Not sure

3. How would you rate their social network (family, work, friends)?

a. Good participation with social networks
b. Adequate participation with social networks
c. Restricted participation with some degree of social isolation
d. Little participation, lonely and socially isolated
e. Not sure

4. How would you rate their financial resources?

a. Financially secure, resources adequate. No identified problems
b. Financially secure, some resource challenges
c. Financially insecure, some resource challenges
d. Financially insecure, very few resources, immediate challenges
e. Not sure

**C. Health Literacy and communication**

1. How well does the patient now understand their health and wellbeing (symptoms, signs or risk factors) and what they need to do to manage their health?

a. Reasonable to good understanding and already engages in managing health or is willing to undertake better management
b. Reasonable to good understanding but do not feel able to engage with advice at this time
c. Little understanding which impacts on their ability to undertake better management
d. Poor understanding with significant impact on ability to manage health
e. Not sure

2. How well do you think your patient can engage in healthcare discussions? (Impairments include deafness, aphasia, alcohol or drug problems, learning difficulties, concentration)

a. Clear and open communication, no identified impairments
b. Adequate communication, with or without minor impairment
c. Some difficulties in communication with or without moderate impairment
d. Serious difficulties in communication, with severe impairment
e. Not sure

**D. Action**

1. Do other services (including health promotion) need to be involved to help this patient?5

a. Other care/services not required at this time
b. Other care/services in place and adequate
c. Other care/services in place but not sufficient
d. Other care/services not in place and required
e. Not sure
